# *Trichinella spiralis* adult excretory-secretory antigen promotes peripheral regulatory T cell differentiation and attenuates experimental colitis via TGF-β-like mechanisms

**DOI:** 10.1186/s13071-025-06877-x

**Published:** 2025-07-01

**Authors:** Xi-Meng Sun, Ze-Ni Luo, Wei Wang, Chun-Yue Hao, Zhi-Ang Li, Saeed El-Ashram, Xin-Ping Zhu

**Affiliations:** 1 Department of Medical Microbiology and Parasitology, School of Basic Medical Sciences, Capital Medical University, Beijing, China; 2 Zoology Department, Faculty of Science, Kafrelsheikh University, Kafr El-Sheikh, Egypt; 3 College of Life Science and Engineering, Foshan University, Foshan, Guangdong Province China

**Keywords:** *Trichinella spiralis*, Adult excretory–secretory antigen, Regulatory T cells, Colitis

## Abstract

**Background:**

*Trichinella spiralis* adult excretory-secretory antigen (*Ts*AES) has been proposed as a potential immunomodulator capable of promoting naïve T cell differentiation into peripheral regulatory T cells (pTregs). This study aims to investigate the effects of *Ts*AES on pTreg development and evaluate its role in alleviating dextran sulfate sodium (DSS)-induced acute colitis.

**Methods:**

Colonic lamina propria mononuclear cells (LPMC) from Foxp3^eGFP^ mice were cultured with interleukin-2 (IL-2) and *Ts*AES for 3 days. The frequency of CD4^+^CD25^+^Foxp3^+^ Treg cells was quantified by flow cytometry. Foxp3^eGFP^ mice subsequently received intraperitoneal injections of *Ts*AES (20 µg/mouse) biweekly for a total of three doses. On day 8 post-model induction, mice were euthanized and colonic tissues collected for immunofluorescence and flow cytometric analysis to assess pTreg populations. Splenic CD4^+^ T cells and dendritic cells (DCs), isolated from Foxp3^eGFP^ mice using magnetic beads, were cultured with IL-2 in either *Ts*AES or phosphate buffered saline for 72 h. The content of IL-10 in culture supernatant was measured by an enzyme-linked immunosorbent assay (ELISA), and CD4^+^Foxp3^+^ Treg cells were sorted and adoptively transferred (40,000 cells/mouse) via tail vein injection into C57BL/6 J mice, followed by DSS administration to induce acute colitis. Body weight was monitored daily, and disease activity index (DAI) was calculated based on fecal characteristics. On day 8, mice were sacrificed, and colons were dissected and subjected to hematoxylin and eosin staining for histopathology study, and flow cytometry was performed to evaluate splenic Treg proportions.

**Results:**

Flow cytometry analysis demonstrated that *Ts*AES stimulation promoted LPMC differentiation into Tregs, leading to a significant increase in pTregs within the colonic lamina propria of *Ts*AES-immunized Foxp3^eGFP^ mice. Although no significant differences in body weight were observed between Treg-infused and non-infused groups, the Treg-infused cohort exhibited a significantly lower DAI. Histological examination showed reduced inflammatory cell infiltration and better-preserved crypt architecture in the colons of Treg-treated mice. The ELISA results showed an elevated IL-10 level in *Ts*AES-treated splenocytes, and flow cytometric analysis confirmed the presence of Tregs in the spleens of recipient mice following adoptive transfer.

**Conclusions:**

*Ts*AES demonstrates tumor growth factor-beta-like activity, driving pTreg differentiation and suppressing DSS-induced colitis with IL-10 upregulation. These findings highlight a beneficial clinical effect of helminth-derived antigens on the immunomodulation and management of inflammatory bowel disease.

**Graphical Abstract:**

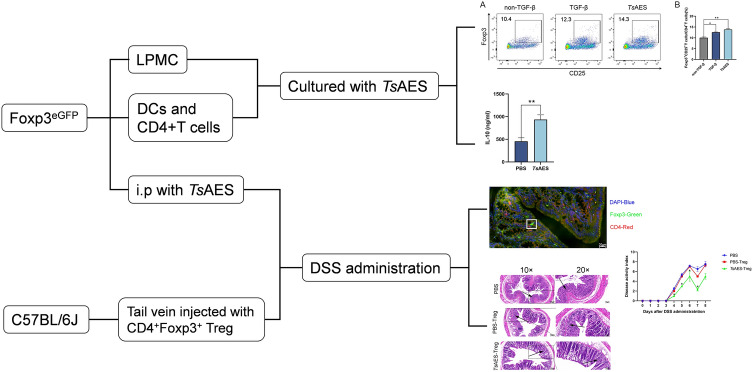

## Background

*Trichinella spiralis* is a parasite that infects both humans and animals, residing primarily in skeletal muscle cells and the small intestine. The infective larval stage of *T. spiralis* inhabits the muscle tissue, while the adult worms reside in the small intestine [1]. Both stages pose significant threats to human and animal health, with severe infections potentially leading to death. Beyond causing trichinosis, research has shown that *Trichinella* spp. can also modulate the host's immune response [2]. Immunoblotting analyses have identified *T. spiralis* antigens, categorizing them into eight groups of muscle larval antigens and one group of adult worm antigens [3, 4]. Extensive studies using rat models have demonstrated that the antigenic epitopes recognized by rodents closely resemble those recognized by humans [5]. *Trichinella spiralis* antigens are generally classified into three types based on their origin: body antigens, surface antigens and excretory-secretory (ES) antigen. Following infection, *T. spiralis* modulates host immunity by stimulating regulatory T cells (Tregs), activating M2 macrophages to secret cytokines and promoting the immune responses of T helper 2 cells (Th2) while suppressing the responses of T helper 1 cells (Th1) typically associated with inflammation [6]. Tregs, together with regulatory B cells (Bregs), activated macrophages and Th2 immune responses, play crucial roles in immunological control during intestinal worm infections [7]. Specifically, intestinal helminth infections can enhance parasite survival while diminishing host immunity by promoting the proliferation of Foxp3^+^ Treg cells [8]. Notably, it has been observed that adult *T. spiralis* stimulate a substantial expansion of Tregs during the early phase of infection [9]. Maizels et al. reported that adult *Brugia malayi* worms excrete and secrete antigens exhibiting tumor growth factor-beta (TGF-β)-like activity, which modulates the host immune response [10]. Numerous studies have confirmed that parasitic infections can induce Treg generation, thereby suppressing host immune responses through direct cell–cell interaction or the secretion of inhibitory cytokines [11].

Tregs have been identified as crucial immune cells in suppressing inflammation. Animal studies have demonstrated that infections with *Heligmosomoides polygyrus* [12], *Schistosoma mansoni* [13] and *Hymenolepis diminuta* [14] can reduce pathological damage associated with colitis, with these protective effects linked to elevated levels of interleukin (IL)-4, IL-10 and TGF-β. For example, infection with *H. polygyrus* leads to a reduction in pro-inflammatory Th1 and Th17 cytokines, accompanied by an increase in anti-inflammatory Treg cells, thereby conferring protection within the intestinal tract [15]. It has been proposed that *H. polygyrus* excretes and secretes antigens that promote the differentiation of naïve T cells into peripheral Tregs (pTregs) through TGF-β-like activity [16], underscoring a critical interaction between the parasite and host immune systems. Adoptive Treg transfer has emerged as a promising therapeutic strategy for intestinal disorders. Specifically, the adoptive transfer of pTregs has been shown to prevent T cell-mediated colitis in mouse model [17]. Moreover, ongoing research aimed at developing safe and effective methods for the adoptive transfer of thymus-derived regulatory T cells (tTregs) for the treatment of human diseases has met with encouraging preliminary results [18].

Building on our data reported in previous publications [19–21], in the current study, the results of which highlight the therapeutic potential of *T. spiralis* excretory-secretory antigens (*Ts*AES) in colitis, we have delved deeper by focusing specifically on the role of pTreg cells and elucidating the underlying mechanisms involved. The results have led us to hypothesize that *Ts*AES possesses TGF-β-like activity and to further determine whether adoptive transfer of pTregs induced by *Ts*AES can alleviate dextran sulfate sodium (DSS)-induced acute colitis.

To address these questions, we initially employed *Ts*AES both in vivo and in vitro to induce the differentiation of intestinal lamina propria cells into Tregs. We then evaluated whether *Ts*AES could similarly promote the differentiation of splenic lymphocytes into Tregs. Using techniques such as flow cytometry and immunofluorescence, we confirmed the presence of TGF-β-like activity within *Ts*AES and then investigated whether the induced Tregs were predominantly pTregs or tTregs. Finally, the *Ts*AES-induced Tregs were adoptively transferred into mice to assess their effectiveness in alleviating DSS-induced acute colitis.

## Methods

### Animals

Female C57BL/6 J mice and female Wistar rats (6–8 weeks old) were purchased from Beijing Vital River Laboratory Animal Technology Co., Ltd (Beijing, China). Female Foxp3^eGFP^ mice (B6. Cg-Foxp3tm2Tch/J, 6–8 weeks old) were purchased from Shanghai Southern Model Biotechnology Co., Ltd. (Shanghai, China). *Trichinella spiralis* used in the study was originally isolated from swine in Heilongjiang Province (ISS 533) and has been maintained in ICR mice. All mice were housed under specific pathogen-free conditions in the Laboratory Animal Services Center of Capital Medical University (Beijing, China) according to the NIH Guidelines for the Care and Use of Laboratory Animals.

### Preparation of *Trichinella* adult excretory secretory antigen

Wistar rats were infected with 12,000 muscle larvae of *T. spiralis* each and then were fasted for 6 days with access to water before being euthanized, and the entire small intestine excised. The intestine was longitudinally opened, rinsed with water and cut into 2- to 3-cm segments. These segments were placed on sterile gauze suspended in a beaker containing preheated saline (37 °C) and incubated at 37 °C in a 5% CO_2_ atmosphere for 4 h. Post-incubation, the liquid was collected for precipitation (30 min), and the supernatant was discarded. The precipitate was washed with sterile saline (2–3 times) and filtered through a 200-mesh sieve. Adult worms were counted microscopically, washed 20 times with saline (0.9% NaCl) containing 2% penicillin–streptomycin (Caisson labs, Logan, UT, USA) and then suspended in RPMI 1640 medium (Thermo Fisher Scientific, Waltham, MA, USA) with 2% penicillin–streptomycin at a concentration of 2000 worms/ml (without serum or phenol red). The culture was then incubated in a 5% CO_2_ incubator at 37 °C for 48 h. After incubation, the culture medium was filtered through a 200-mesh sieve and centrifuged at 1000 *g* for 5 min at 4 °C; the supernatant was discarded and the remaining filtrate was transferred to a 3 kDa ultrafiltration tube (Millipore Amicon Ultra-15, NMWL 3000; MilliporeSigma, Merck, Burlington, MA, USA) and centrifuged at 2400 *g* for 50 min at 4 °C. Sterile phosphate-buffered saline (PBS; 5 ml) was added and replaced 4–5 times [22]. Protein concentration in the AES was determined using the BCA protein assay (Merck, Darmstadt, Germany). Finally, *Ts*AES was aliquoted into 200-µl tubes and stored at − 80 °C.

### Intraperitoneal injection of *Ts*AES and establishment of acute colitis model

*Trichinella* adult excretory-secretory antigen (20 µg/mouse) was injected intraperitoneally into Foxp3^eGFP^ mice every 2 weeks for a total of three injections. The mice were allowed free access to the 3% DSS polymer solution (36,000–50,000 MW; MP Biomedicals, Solon, OH, USA) for 6 days after the last injection and then free access to water for 2 days. During the model's development, the body weight of each mouse was recorded every morning at 9 a.m., the stool properties and blood in the stool were observed and the corresponding blood stool scores were assigned according to Table 1. The mice were euthanized on day 8 after the acute colitis model was established, and the colon was dissected and collected.

**Table 1:** Disease activity index criteria

Weight loss (%)	Stool properties^a^	Blood stool score
0	Normal	0
1 ~ 5	Slightly loose	1
6 ~ 10	Loose, soft, slightly bloody stools	2
11 ~ 15	Diarrhea	3
> 15	Watery stool, severe bloody stool	4

^a^Normal stool: formed stool; loose stool: mushy, semi-formed stool that does not adhere to the anus; loose stool: watery stool that can adhere to the anus

### Scoring criteria for the acute colitis model in mice

The mice were weighed every day at 9:00 a.m. at the start of the acute colitis model, and the fecal conditions (bloody stools, diarrhea, increased number of stools, etc.) were observed, and the disease activity index (DAI) was calculated using the international common scale (Table 1) as previously described [1]. Total DAI = 12 points (weight loss score + fecal trait score + bloody stool score).

### Isolation of mouse colonic lamina propria mononuclear cells

The colons were excised and placed in Ca^2^⁺/Mg^2^⁺-free Hank’s balanced salt solution (HBSS; Gibco, Thermo Fisher Scientific, Carlsbad, CA, USA). Colonic lamina propria mononuclear cells (LPMCs) were isolated using the Lamina Propria Dissociation Kit (Miltenyi Biotec GmbH, Cologne, Germany) in a gentleMACS Octo Dissociator (Miltenyi Biotec GmbH) at 37 °C [18]. In brief, the mice were sacrificed, the colon was removed intact and the fat and the mesentery were removed and cleaned from each colon. The colon was then opened longitudinally and placed in a 50-ml tube containing 20 ml PBS, with shaking on an oscillator until clean. The cleaned colon was then cut into 0.5-cm-long segments and incubated in predigestion solution containing Ca^2^⁺/Mg^2^⁺-free HBSS (1 mM dithiothreitol [DTT; Absin, Shanghai, China], 5 mM EDTA [Calbiochem, Merck, Darmstadt, Germany], 5% fetal bovine serum [FBS; Gibco, Thermo Fisher Scientific], 100 U/ml penicillin, 100 U/ml streptomycin and 10 mM HEPES [Absin, Shanghai, China]) for 60 min, under continuous rotation at 37 °C to remove epithelial layers. This step was repeated with a new predigestion solution until the tissue was clear. The predigested colon fragments were washed in Ca^2^⁺/Mg^2^⁺-free HBSS to remove the EDTA. The remaining colon pieces were dried and transferred to a tissue processing tube, followed by digestion with an enzyme mix from the Lamina Propria Dissociation Kit (Miltenyi Biotec GmbH) for 30 min at 37 °C. The suspension was filtered through a cell sieve (pore size: 40-µm), and the single-cell suspension was washed once with PBS, following which the PBS was replaced with culture medium.

### Incubation of LPMCs with *Ts*AES

Lamina propria mononuclear cells were isolated, counted and the concentration adjusted to 3 × 10^6^ cells/ml. Complete medium (RPMI 1640, 10% FBS, 2% penicillin–streptomycin) was supplemented with 1 µg of anti-CD3 soluble antibody, 2 µg of anti-CD28 soluble antibody and 100 U/ml of IL-2 following cell transplantation. The experimental group received 6 µg/ml of *Ts*AES, while the control group was treated with an equivalent volume of PBS. Cells were incubated at 37 °C in a 5% CO_2_ atmosphere for 72 h. After incubation, cells were transferred to a 15-ml tube and centrifuged at 500 *g* for 6 min at 4 °C, following which the pellet was collected and the supernatant discarded.

### Isolation of CD4^+^T cells and DCs

Cell counting was performed on spleen lymphocytes obtained from normal Foxp3^eGFP^ mice. The Mouse CD4+ T Cell Isolation Kit (Miltenyi Biotec) was used to isolate CD4⁺ T cells, and the cell concentration was then adjusted to 10^7^ cells/ml. Subsequently, 40 µl of sorting buffer (PBS, 0.5% bovine serum albumin [BSA], 2 mM EDTA) and 10 µl of a biotin-antibody cocktail were added to the CD4⁺ T cell suspension, which was then thoroughly mixed before being incubated at 4 °C for 5 min. A mixture of sorting buffer (30 µl) and anti-biotin magnetic beads (20 µl) was prepared and stored at 4 °C for 10 min. The Miltenyi Biotec LS column was magnetized and placed on a magnetic stand, followed by washing with 3 ml of sorting buffer. The cell-containing suspension was added to the LS column, and unlabeled CD4⁺ T cells were collected in a 15-ml tube from the flow-through.

For dendritic cell (DC) isolation, the cell concentration was adjusted to 10^8^ cells/ml. A total of 400 µl of sorting buffer (PBS, 0.5% BSA, 2 mM EDTA) and 100 µl of CD11c-labeled magnetic beads were added to the DC solution and mixed thoroughly, then incubated at 4 °C in the dark for 10 min. Sorting buffer (10 ml) was then added, and the mixture was centrifuged at 500 *g* for 6 min at 4 °C. The supernatant was carefully discarded, and the cells were resuspended in 500 µl of sorting buffer. The LS column was magnetized and placed on the magnetic stand, followed by washing with 3 ml of sorting buffer. After the cell suspension was added to the column, unlabeled cells were collected in a 15-ml tube. The column was rinsed with an appropriate amount of sorting buffer. Finally, the LS column was removed from the magnet and placed in a 15-ml tube containing 5 ml of sorting buffer, and the plunger was firmly pushed into the LS column to elute the magnetically labeled DCs.

### Co-cultivation of CD4^+^ T cells and DC cells

CD4⁺ T cells and DCs were mixed at a ratio of 1:2, with the final cell concentration adjusted to 10^6^ cells/ml. The cell suspension was transferred to complete medium (RPMI 1640, 10% FBS, 2% penicillin–streptomycin) and supplemented with 1 µg of anti-CD3 soluble antibody, 2 µg of anti-CD28 soluble antibody and 100 U/ml IL-2. The suspension was then divided equally into several culture dishes: 6 µg/ml of *Ts*AES, 2 µl/ml of lipopolysaccharide (LPS; Aldrich-Sigma, St. Louis, MO, USA), 6 µg/ml of TGF-β (Sino Biological, Beijing, China) and an equivalent volume of PBS. Both dishes were incubated at 37 °C with 5% CO_2_ for 72 h. Following incubation, cells were transferred to a 15-ml tube, centrifuged at 500 *g* for 6 min at 4 °C; the resulting pellet was collected separately. An appropriate volume of PBS was added for resuspension of the pellet and cell counting. The supernatant was aliquoted and stored at − 80 °C for further analysis.

### Treg adoptive transfer

C57BL/6 J mice were injected via the tail vein with in vitro-cultured Treg cells (40,000 cells/mouse). Starting 3 days post-injection, the mice were allowed free access to 3% DSS solution for 6 days, followed by a 2-day period of free access to drinking water. Throughout the development of the model, body weight was recorded daily at 9 a.m., and stool consistency and the presence of blood in the stool were monitored, with corresponding scores assigned according to Table 1. On day 8 following the establishment of the acute colitis model, the mice were euthanized by cervical dislocation, and the colon and spleen were collected for further analysis.

### Flow cytometry

The following reagent and antibodies were purchased from eBioScience, Inc., Thermo Fisher Scientific (San Diego, CA, USA) and used in the flow cytometry analysis: blocking antibody Anti-CD16/32, PerCP-eFluor710-labeled anti-CD3 antibody, PE-labeled anti-CD4 antibody, APC-labeled anti-CD25 antibody, fixation nucleating agent, permeation buffer, PE-Cy7-labeled anti-Helios antibody and FITC-labeled anti-Foxp3 antibody.

The cell concentration was adjusted to 2 × 10^6^ cells per 100 µl using PBS buffer. To detect dead cells, the suspended cells were pre-incubated with the Zombie Violet Fixable Viability Kit (BioLegend, San Diego, CA, USA). Approximately 2 µl of the blocking antibody Anti-CD16/32 (clone 93; eBioscience, Thermo Fisher Scientific) was added to 2 × 10^6^ cells and incubated for 20 min at 4 °C, following which the cells were incubated with 1.25 µl of PerCP-eFluor710-labeled anti-CD3 antibody, 0.4 µl of PE-labeled anti-CD4 antibody and 0.625 µ; of APC-labeled anti-CD25 antibody at 4 °C for 30 min. After antibody incubation, the cells were rinsed with 5 ml of PBS buffer and centrifuged at 500 *g* for 6 min at 4 °C. The pellet was resuspended, and cells were fixed and permeabilized using the BD Cytofix/Cytoperm™ Fixation/Permeabilization Kit (BD Bioscience, Franklin Lakes, NJ, USA) according to the manufacturer’s instructions. For staining of the nucleus, 2.5 µl of PE-Cy7-labeled anti-Helios antibody and 0.03 µl of FITC-labeled anti-Foxp3 antibody were added to the cells and incubated at room temperature for 50 min. The cells were rinsed with 5 ml of permeabilization buffer, centrifuged at 500 *g* for 5 min at room temperature and resuspended.

To prepare single-stained samples for compensation adjustments, 1.25 µl of PerCP-eFluor710-labeled anti-CD3 antibody, 1.25 µL of PE-labeled anti-CD4 antibody, 0.625 µL of APC-labeled anti-CD25 antibody, 0.03 µL of FITC-labeled anti-Foxp3 antibody and 2.5 µL of PE-Cy7-labeled anti-Helios antibody were used. Compensation adjustments were performed after detection of the samples and single-stained controls using the Cytek Aurora Spectral Flow Cytometer (equipped with SpectroFlo version 2.2.0) (Cytek Biosciences, San Jose, CA, USA). Data analysis was conducted using FlowJo software (Tomy Digital Biology, Tokyo, Japan).

### Detection of IL-10 in cell culture supernatant by enzyme-linked immunosorbent assay

Single cell suspensions of splenocytes were prepared in mouse lymphocyte separation medium according to the manufacturer’s instructions (Dakewei Biotech, Shenzhen, China). Subsequently, 2 × 10^5^/well splenocytes in 200 μl of RPMI-1640 media containing 10% FBS; Life Technologies, Thermo Fisher Scientific, Carlsbad, CA, USA) and 100 U of penicillin/streptomycin/ml were cultured with *Ts*AES, and PBS in 96-well plates at 37 °C, 5% CO_2_ for 72 h. Culture supernatants were recovered for detecting IL-10 using the Mouse IL-10 enzyme-linked immunosorbent assay (ELISA) Kit (Dakewei Biotech, Shenzhen, China) according to the manufacturer’s instructions.

### Hematoxylin and eosin staining of paraffin-embedded colon sections

Fresh colon tissue segments (length: 1 cm) were fixed in 4% paraformaldehyde for a minimum of 24 h, trimmed in a fume hood and dehydrated through a series of ethanol concentrations (75%, 85%, 90%, 95%) and xylene treatments. Post-dehydration, the tissue was embedded in paraffin, cooled and sectioned into 4-μm-thick sections. The sections were flattened, mounted on slides and baked to remove moisture. Deparaffinization involved xylene and ethanol rinses, followed by hematoxylin and eosin (H&E) staining with intermediate rinses and differentiation steps. The sections were then dehydrated again, cleared with xylene and toluene and mounted with neutral gum for microscopic analysis.

### Immunofluorescence of colonic sections

Colonic tissue sections were obtained from Foxp3^eGFP^ reporter mice and dewaxed through sequential treatments with xylene and ethanol solutions, followed by washing with distilled water. Antigen retrieval was achieved using EDTA buffer and microwave heating, with subsequent PBS rinses. Tissue sections were marked and blocked with BSA to minimize non-specific binding before incubation with rabbit anti-CD4 mAb (Servicebio, Wuhan, China) at a 1:400 dilution overnight at 4 °C. Post-incubation, sections were washed and incubated with Cy3-conjugated goat anti-rabbit immunoglobulin G secondary antibody for 50 min at room temperature. DAPI (1:5000 dilution; Sigma-Aldrich) staining was performed for nuclear visualization, followed by additional PBS rinses and treatment with an autofluorescence quencher. Finally, the sections were mounted with an anti-fade medium for fluorescence microscopy analysis.

### Statistical analysis

The experimental data were analyzed using GraphPad Prism 8 software (GraphPad Software, San Diego, CA, USA). Unpaired two-tailed Student’s t-test was used to compare two groups, and one-way analysis of variance (ANOVA) with the Bonferroni correction was used to compare multiple groups. Data are expressed as the mean ± standard error of the mean (SEM). For the difference to be statistically significant, *P* < 0.05 was utilized as the threshold.

## Results

### *Ts*AES exhibits TFG-β-like activity

**Fig. 1:**
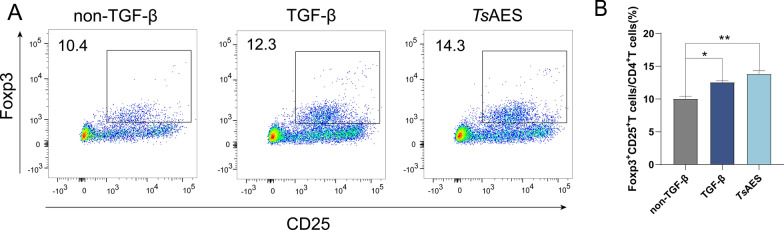
*Ts*AES promotes naïve T cells into regulatory T cells (Tregs). Dendritic cells (DCs) and CD4+ T cells were stimulated with *Ts*AES, TGF-β or without of TGF-β for 72 h. **A** Flow cytometry was used to analyze the expression levels of CD25^+^Foxp3^+^ Tregs stimulated by *Ts*AES, TGF-β or the absence of TGF-β. **B** The percentage of CD25^+^Foxp3 ^+^T cells to CD4^+^T cells under different stimulation conditions. Data are expressed as means ± standard error of the mean from 2 independent experiments, *n* = 5. Asterisks indicate a significant difference in the proportion of CD4^+^CD25^+^Foxp3^+^ Tregs to CD4^+^ T cells between the TGF-β and *Ts*AES groups and the non-TGF-β group at *P < 0.05 and **P < 0.01. TGF-β, tumor growth factor-beta;* Ts*AES, *Trichinella* adult excretory-secretory antigen

**Fig. 2:**
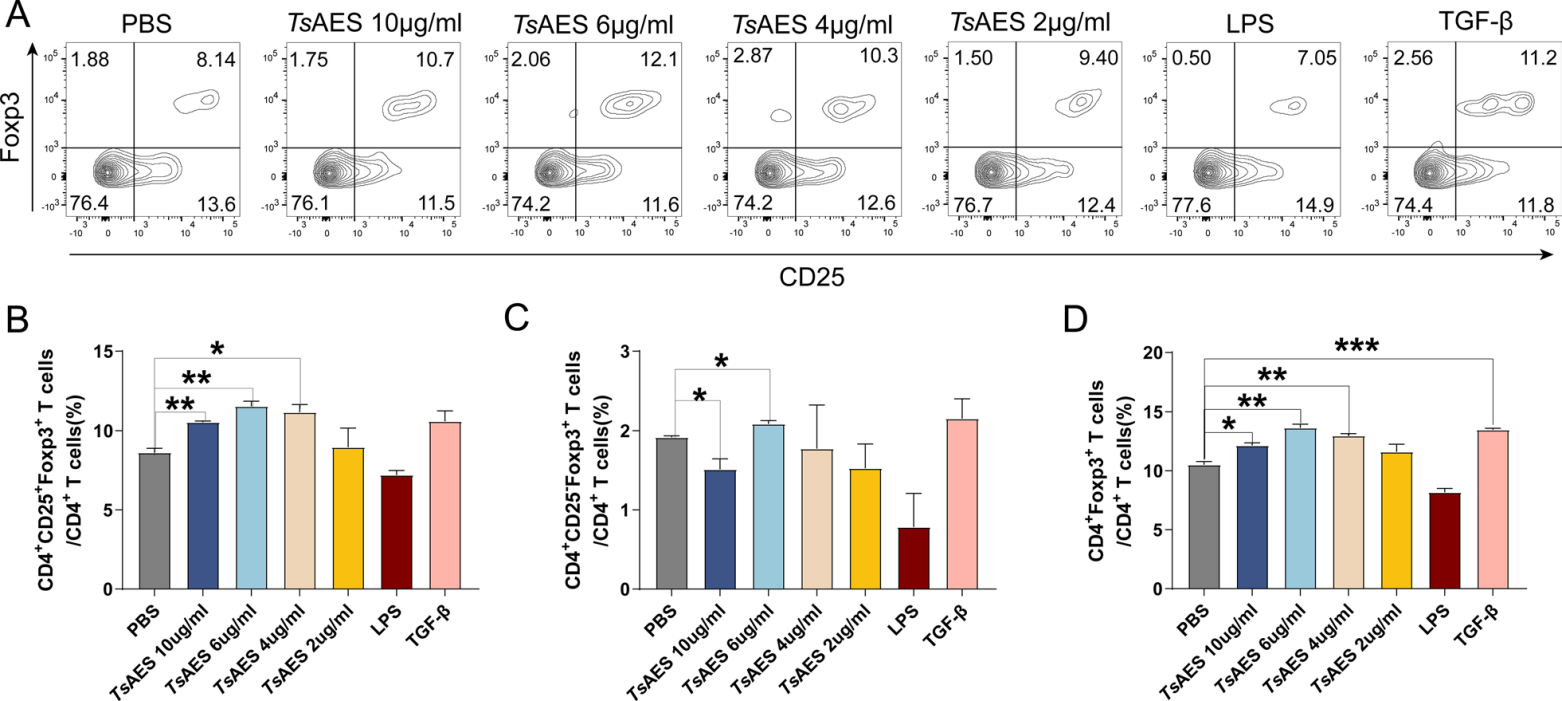
*Ts*AES induces colonic lamina propria mononuclear cells (LPMCs) to differentiate into regulatory T cells (Tregs). LPMCs extracted from Foxp3^eGFP^ mice were cultured in PBS, LPS, TGF-β or different concentrations of *Ts*AES for 72 h. **A** Flow cytometry was used to analyze the expression levels of CD25^+^Foxp3^+^Tregs under different stimulation conditions. **B** The percentage of CD4^+^CD25^+^Foxp3^+^ T cells to CD4^+^ T cells. **C** The percentage of CD4^+^CD25^−^Foxp3^+^ T cells to CD4^+^ T cells. **D** The percentage of CD4^+^ Foxp3^+^ T cells to CD4^+^ T cells. Data are expressed as the mean ± standard error of the mean from 2 independent experiments, *n* = 5. Asterisks indicate a significant difference in the ratio of CD4^+^CD25^+^Foxp3^+^ Tregs, CD4^+^CD25^−^Foxp3^+^ Tregs and CD4^+^Foxp3^+^ Treg cells to CD4^+^ T cells between each experimental group and PBS group at *P < 0.05, **P < 0.01, ***P < 0.001. LPS, Lipopolysaccharide; PBS, phosphate-buffered saline; TGF-β, tumor growth factor-beta;* Ts*AES, *Trichinella* adult excretory-secretory antigen

Dendritic cells and CD4^+^ T Cells were cultured with IL-2, and then stimulated with *Ts*AES, TGF-β or the absence of TGF-β for 72 h. Flow cytometry was then used to quantify the proportion of CD4^+^CD25^+^Foxp3^+^ Treg cells. The results indicated that stimulation with TGF-β and *Ts*AES led to the differentiation of Tregs from naïve T cells, with *Ts*AES inducing a higher percentage of Tregs compared to TGF-β stimulation (Fig. 1). LPMCs extracted from Foxp3^eGFP^ mice were cultured in PBS, LPS, TGF-β or different concentrations of *Ts*AES for 72 h. Notably, LPMCs exhibited the significant effect of producing Tregs following stimulation with the 6 μg/ml concentration of *Ts*AES (Fig. 2). These findings indicated that *Ts*AES possesses TGF-β-like activity.

### *Ts*AES increases the number of pTregs (Helios-Tregs) in colonic lamina propria

**Fig. 3:**
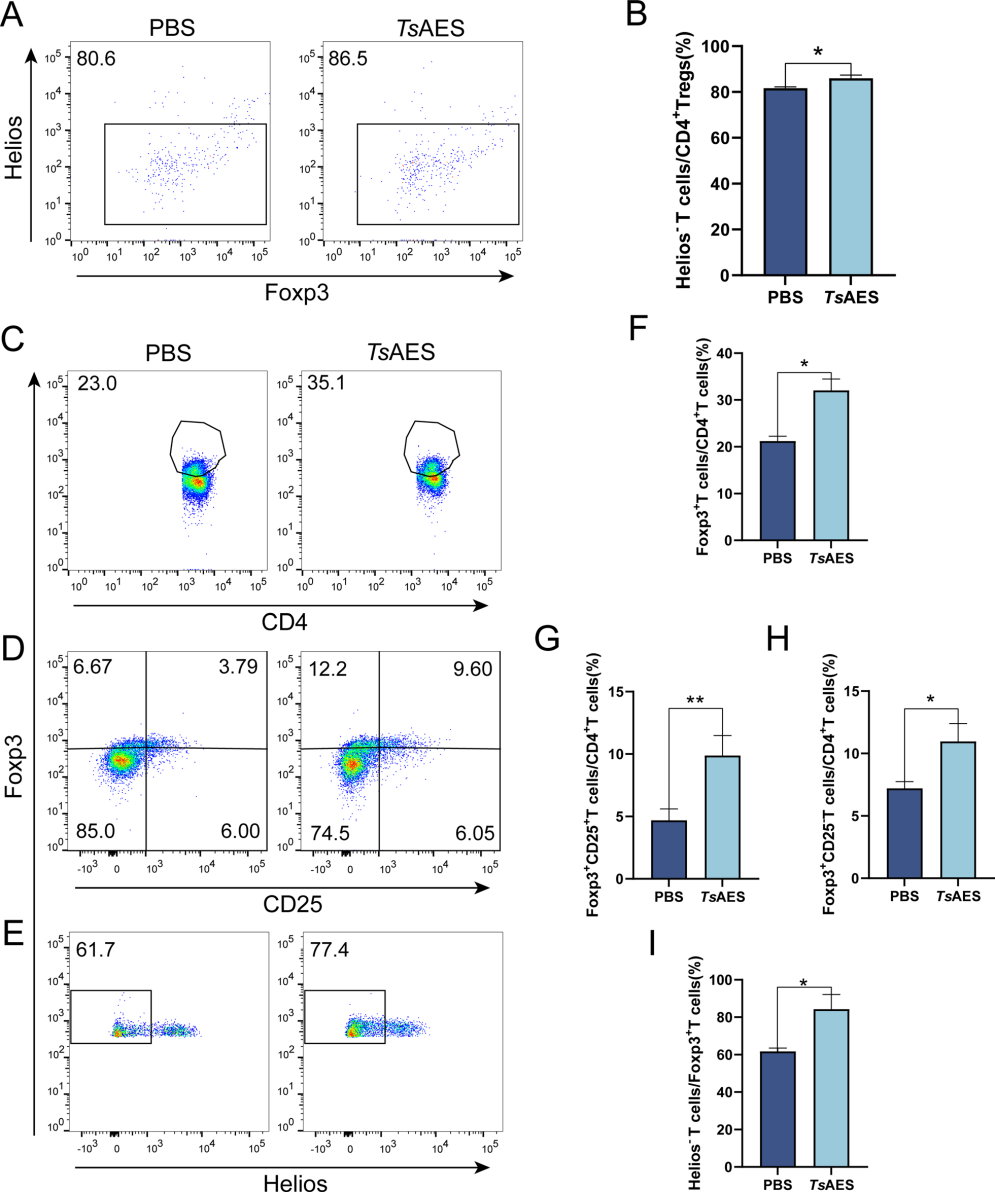
*Ts*AES increases colonic lamina propria (cLP) peripheral regulatory T cells (pTregs) in vitro and in vivo. Lamina propria mononuclear cells (LPMCs) were cultured under various *Ts*AES conditions and compared to those cultured in PBS. **A** Flow cytometry was used to analyze the expression levels of CD4^+^Foxp3^+^Helios^−^ T cells in the PBS and *Ts*AES groups. **B** Percentage of CD4^+^Foxp3^+^Helios^−^ T cells to CD4^+^ Treg cells. To further assess the effects of *Ts*AES in vivo, each Foxp3^eGFP^ mouse received *Ts*AES or PBS before dextran sulfate sodium (DSS) administration, following which the colonic LPMCs were harvested. **C** Flow cytometry was used to analyze the expression levels of CD4^+^Foxp3^+^ T cells in the PBS and *Ts*AES groups. **D** Expression levels of CD25^+^Foxp3^+^ T cells in the PBS and *Ts*AES groups. **E** Expression levels of Foxp3^+^Helios^+^ T cells in the PBS and *Ts*AES groups. **F** Percentage of CD4^+^Foxp3^+^T cells to CD4^+^T cells. **G** Percentage of CD25^−^Foxp3^+^T cells to CD4^+^T cells. **H** Percentage of CD25^+^Foxp3^+^T cells to CD4^+^T cells. **I** Percentage of Helios^−^Foxp3^+^T cells to Foxp3^+^T cells. Data are expressed as the mean ± standard error of the mean from 2 independent experiments, *n* = 5. Asterisks indicate significant differences among the proportions of CD4+ CD25^+^Foxp3^+^ Tregs, CD4+ CD25^−^Foxp3^+^ in CD4^+^ Tregs and Foxp3^+^ Helios^−^ Treg in CD4^+^Foxp3^+^ Tregs in each group at *P < 0.05, **P < 0.01. PBS, Phosphate-buffered saline;* Ts*AES, *Trichinella* adult excretory-secretory antigen

Lamina propria mononuclear cells isolated from Foxp3^eGFP^ mice were cultured with PBS or *Ts*AES for 72 h. Flow cytometry staining was performed using CD3, CD4, CD25, Helios and Foxp3 antibodies. CD4^+^Foxp3^+^Helios^−^ Tregs represent pTregs, while CD4^+^Foxp3^+^ Helios^+^ Tregs correspond to tTregs. Flow cytometric analysis revealed an increased ratio of CD4^+^Foxp3^+^Helios^−^ Tregs among CD4^+^ Tregs in LPMCs cultured with *Ts*AES compared with the PBS group (Fig. 3a, b).

To further evaluate the effects of *Ts*AES in vivo, each Foxp3^eGFP^ mouse was administered intraperitoneal injections of 20 µg *Ts*AES at 2-weeks intervals for a total of three doses prior to 3% DSS administration. Mice were then euthanized to harvest colonic LPMCs. Flow cytometric analysis demonstrated an increase in the proportions of both CD4^+^CD25^+^Foxp3^+^ Tregs (Fig. 3c, d, f, h) and CD4^+^CD25^−^Foxp3^+^ Tregs (Fig. 3d, g) to CD4^+^ Tregs, as well as an elevation in CD4^+^Foxp3^+^Helios^−^ Tregs (Fig. 3e, i) among CD4^+^Foxp3^+^ Tregs.

**Fig. 4:**
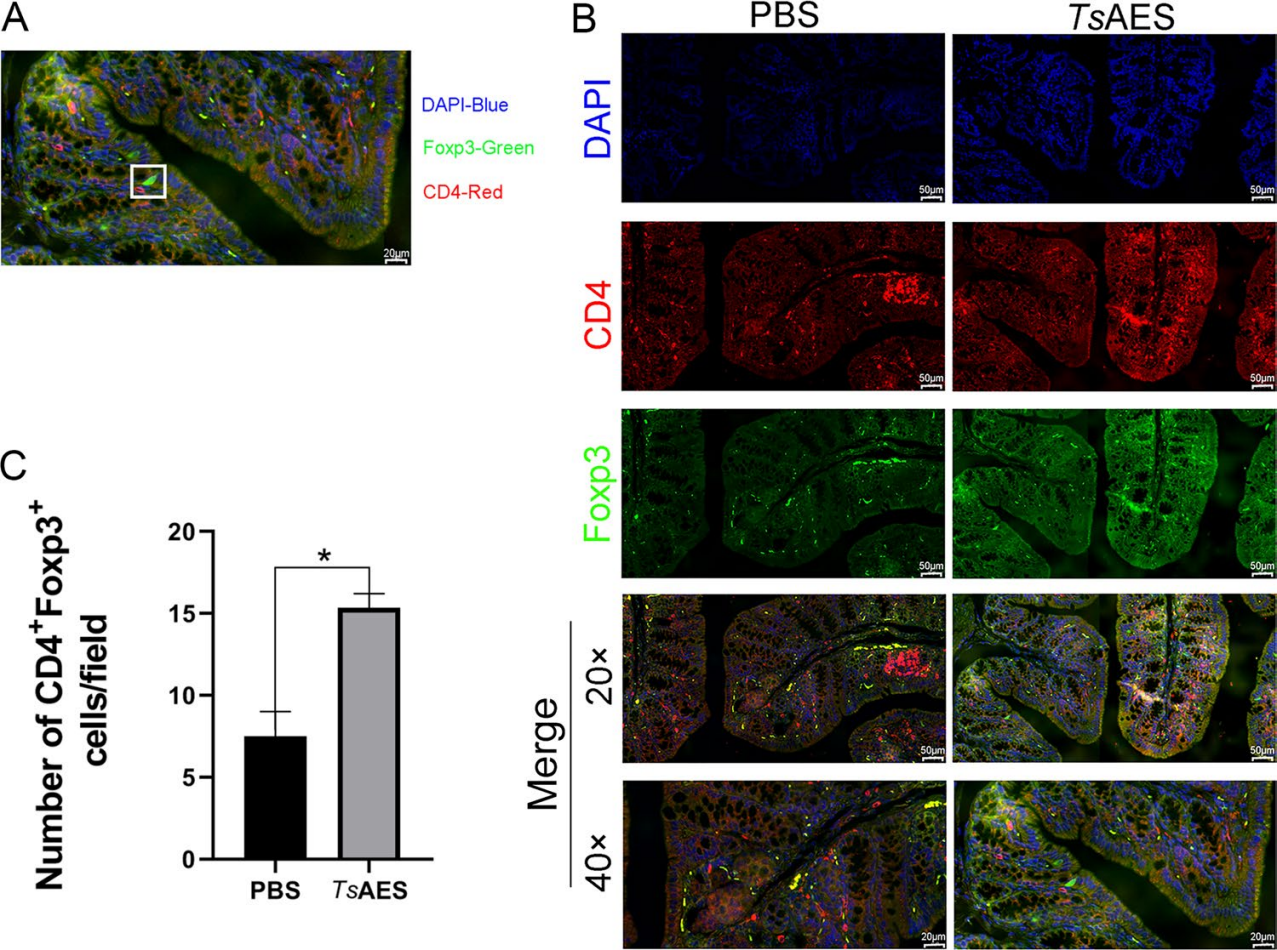
Treatment with *Ts*AES increased CD4^+^Foxp3^+^ regulatory T cells (Tregs) in the colon cross-sections. Each Foxp3^eGFP^ mouse received either *Ts*AES or PBS prior to the administration of dextran sulfate sodium (DSS), following which the proximal colon was harvested. **A** Immunofluorescence staining of the colon cross-section with antibodies for CD4 (red), counterstained with DAPI (blue) and spontaneous FITC fluorescence in Foxp3^eGFP^ mice. **B** Colon sections of mice from different groups. Illustrations are representative of 5 mice per group. **C** Number of CD4^+^Foxp3^+^Treg cells in the PBS or *Ts*AES group. Data are expressed as the mean ± standard error of the mean from 2 independent experiments. Asterisk indicates that the number of Treg cells produced by in vivo immunization with *Ts*AES is significantly different from that of mice injected with PBS at *P < 0.05. PBS, Phosphate-buffered saline;* Ts*AES, *Trichinella* adult excretory-secretory antigen

The proximal colon was excised, fixed, embedded and sectioned for immunofluorescence analysis (with CD4 stained red using Cy3 and nuclei stained blue with DAPI) to visualize CD4^+^Foxp3^+^ Tregs in cross-sections of the colon (Fig. 4a, b). A fully automated slide scanner was employed to quantify Foxp3^+^ Treg cells within the colon cross-sections. The results indicated that the number of Foxp3^+^ Treg cells was significantly higher in the *Ts*AES group compared to the PBS group (Fig. 4c).

Collectively, these findings suggested that *Ts*AES may enhance the proportion of pTreg cells originating from peripheral tissues within the colonic lamina propria (cLP).

### *Ts*AES upregulates the level of cytokine IL-10 secreted by lymphocytes

**Fig. 5:**
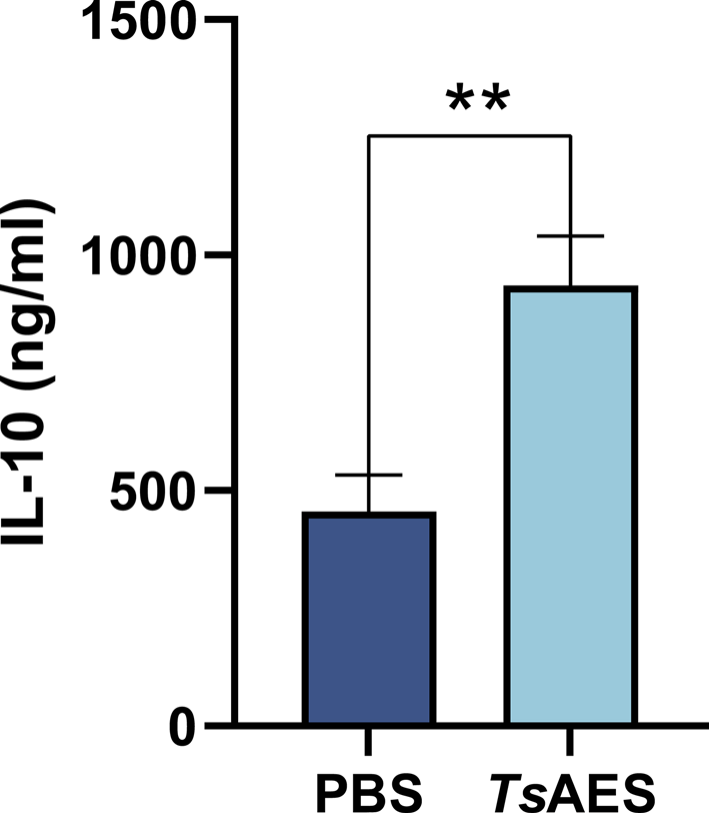
The levels of cytokine interleukin (IL)-10 were determined using an enzyme-linked immunosorbent assay. Data are expressed as the mean ± standard error of the mean from 2 independent experiments, *n* = 5. Asterisks indicate that the content of cytokine IL-10 in the cell supernatant of the *Ts*AES group is significantly different from that of the PBS group at **P < 0.01. IL, Interleukin; PBS, phosphate-buffered saline;* Ts*AES, *Trichinella* adult excretory-secretory antigen

Splenic CD4+ T cells isolated from Foxp3^eGFP^ mice were co-cultured with IL-2 and DCs in the presence of *Ts*AES or PBS for 72 h. Analysis of the culture supernatants using the ELISA demonstrated a significant elevation in IL-10 levels in the *Ts*AES group compared to the PBS group (Fig. 5). These findings indicated that *Ts*AES enhances the secretion of the cytokine IL-10 by lymphocytes.

### Adoptive transfer of Tregs increases the number of splenic Tregs

**Fig. 6:**
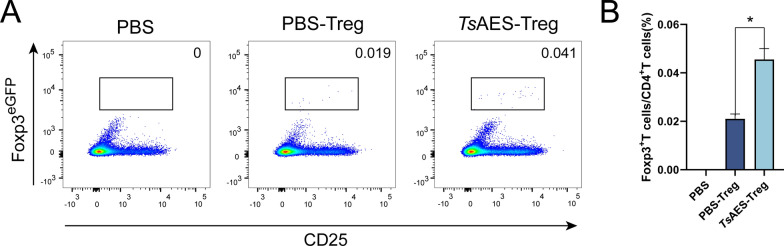
Increased splenic Tregs in adoptively transferred Treg mice. **A** Flow cytometry was used to analyze the expression levels of CD25^+^Foxp3^+^ T cells in different experimental groups. **B** Expression levels of CD25^+^Foxp3^+^ T cells in the different experimental groups. Data are expressed as the mean ± standard error of the mean from 2 independent experiments, *n* = 5. Asterisk indicates that the proportion of Treg cells in CD4^+^ T cells after adoptive transfer in the *Ts*AES-Treg group is significantly different from that in the PBS-Treg group at *P < 0.05. PBS, phosphate-buffered saline; Tregs, regulatory T cells;* Ts*AES, *Trichinella* adult excretory-secretory antigen

To assess the beneficial clinical effect of *Ts*AES-primed Tregs, in vitro-generated Foxp3^eGFP^ Tregs were adoptively transferred into C57BL/6 J mice via the tail vein (40,000 cells/mouse) before 3% DSS administration. The mice were divided into three groups: PBS control, PBS-Treg (Tregs cultured with PBS) and *Ts*AES-Treg (Tregs cultured with *Ts*AES). On the ninth day of post-model establishment, splenic Treg populations were quantified. Flow cytometry results indicated that the Treg cells generated in vitro were successfully detected in both of the Treg groups that received them, with the *Ts*AES-Treg group exhibiting a significantly higher number compared to the PBS-Treg group (Fig. 6).

### Adoptive transfer of Treg alleviates DSS-induced acute colitis

**Fig. 7:**
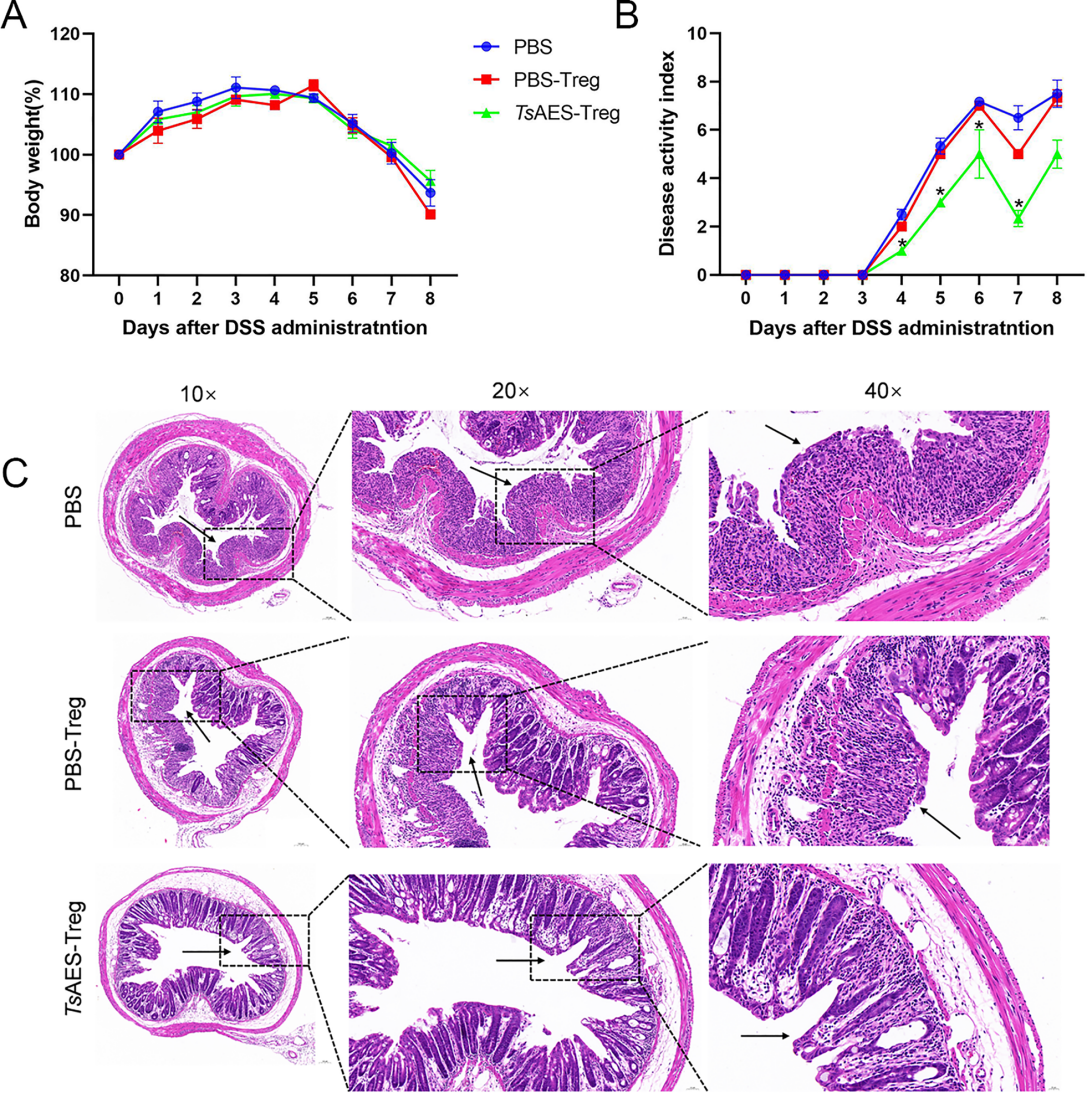
Adoptive transfer of Tregs alleviates body weight, disease activity index (DAI) and colonic pathological damage of dextran sulfate sodium (DSS)-induced acute colitis mice. **A** Body weight changes (relative to initial weight). **B** Changes in total DAI. **C** Representative histopathological sections of colon at magnifications of 10×, 20× and 40×, respectively . Data are expressed as the mean ± standard error of the mean from 2 independent experiments, *n* = 5. Asterisk indicates a significant difference in mouse body weight and DAI between the *Ts*AES-Treg experimental group and the PBS control group at *P < 0.05. PBS, phosphate-buffered saline; Tregs, regulatory T cells;* Ts*AES, *Trichinella* adult excretory-secretory antigen

C57BL/6 J mice received tail vein injections of in vitro-cultured Treg cells (stimulated with *Ts*AES or PBS), or PBS alone. Body weight and fecal conditions were monitored daily, and the related scores were summarized (see Table 1). All groups exhibited dramatic weight loss beginning on day 5. While the *Ts*AES-Treg group exhibited slightly higher body weights on days 7 and 8 compared to the other two groups, no statistically significant differences were noted (Fig. 7a). Notably, the reinfused *Ts*AES-Treg group displayed a reduced DAI compared to the other groups (Fig. 7b).

Histological examination of the colon revealed numerous inflammatory cells in the PBS group, characterized by the infiltration of neutrophils, lymphocytes and plasma cells, and accompanied by evident tissue necrosis. The glandular crypt structure was disrupted, exhibiting deformities and cystic dilation, with significant reductions or complete loss of crypts (Fig. 7c). The PBS-Treg group demonstrated similar findings, with extensive damage to the glandular crypts and a marked reduction in goblet cells, alongside a significant infiltration of inflammatory cells. In contrast, the *Ts*AES-Treg group showed less colonic mucosal damage, a more intact epithelial structure, well-formed glandular crypts and reduced infiltration of inflammatory cells. These observations highlighted the protective effects of *Ts*AES-induced Treg in DSS-induced colonic injury.

## Discussion

The intricate interplay between helminth-derived immunomodulators and host Treg responses has emerged as a critical research focus in mucosal immunology. Our findings demonstrate that *Ts*AES activates a TGF-β-dependent pathway to selectively expand pTregs, thereby mitigating DSS-induced colitis. This observation not only aligns with but also extends existing paradigms of helminth-mediated immune regulation. While previous studies have underscored the therapeutic potential of *Ts*AES in colitis, the present investigation advances the field by specifically elucidating the role of pTreg cells and providing a comprehensive analysis of the underlying mechanisms involved.

The utilization of Foxp3^eGFP^ mice combined with techniques such as flow cytometry and immunofluorescence enabled a detailed analysis of Treg populations within the colon, providing novel new insights into this area of research. Previous studies have evaluated the efficacy of *Ts*AES in alleviating DSS-induced inflammatory colitis in mice by examining various parameters, including the DAI, colon length, histological inflammation and cytokine profiles [19]. The results of these studies demonstrated that *Ts*AES treatment significantly reduces colitis severity, likely through the downregulation of pro-inflammatory cytokines such as interferon-gamma (IFN-γ), IL-6 and IL-17, together with the upregulation of regulatory cytokines such as IL-10 and TGF-β, as well as an increase in Treg generation, particularly within mesenteric lymph nodes. In contrast, in the current study, we specifically investigated the role of *Ts*AES in inducing pTreg cells and its consequent effect on DSS-induced acute colitis in mice. Our findings revealed that *Ts*AES exhibits TGF-β-like activity, promoting the differentiation of naive T cells into pTreg cells, which contributes to the amelioration of acute colitis. This is corroborated by observed reductions in the DAI scores and improvements in histopathological outcomes in treated mice. Notably, *Ts*AES also stimulated T cell differentiation in vitro under two distinct conditions: concanavalin A (ConA) and anti-CD3 (α-CD3) stimulation. The induction effect was markedly stronger under α-CD3 stimulation compared to ConA stimulation, with LPS interference effectively eliminated, underscoring the specificity of *Ts*AES in driving pTreg differentiation.

In the present study, *Ts*AES was demonstrated to enhance Treg cell production across multiple experimental settings, highlighting its ability to induce TGF-β-like effects both in vitro and in vivo. Inflammatory bowel disease (IBD), which includes Crohn's disease (CD) and ulcerative colitis (UC), is characterized by chronic inflammation of the gastrointestinal tract [23, 24]. The progression of IBD is influenced by various factors, such as genetic predisposition, gut microbiota dysbiosis and immune system dysregulation. This dysregulation results in elevated levels of pro-inflammatory cytokines and an imbalanced T cell response, characterized notably by an increase in Th17 cells and a decrease in Tregs [25].

Tregs play a crucial role in maintaining intestinal homeostasis and are primarily composed of two major subtypes: thymus-derived Tregs (tTregs) and peripheral Tregs (pTregs). tTregs develop in the thymus in response to self-antigens, whereas pTregs arise from mature peripheral T cells upon continuous stimulation by exogenous antigens, a process mainly driven by cytokines such as IL-2 and TGF-β. Within the cLP, both tTregs and pTregs coexist and are essential for preserving immune balance. Research has shown that a reduction in Treg numbers or impairment of their function in the murine intestine can lead to the development of colitis [26]. Additionally, antigens excreted and secreted by *H. polygyrus* have been found to exhibit TGF-β-like activity, thereby modulating Treg differentiation [16]. Moreover, adoptive transfer of pTregs has been demonstrated to prevent colitis in mouse models, further underscoring the therapeutic potential of Treg-based approaches [17].

Our findings align with those of Grainger et al. [16], who demonstrated that rodents infected with *H. polygyrus* and exposed to its excretory-secreted antigen (HES) exhibited increased levels of Treg cells. These authors employed HES to induce CD4^+^ T cells and analyzed Helios and Foxp3 expression via flow cytometry, confirming the expansion of Helios^−^ Tregs. Their results validated the TFG-β-like activity of HES through Th17 induction experiments [16]. In our study, *Ts*AES was similarly used to induce Th17 cells; however, potential contamination during the culture process led to reduced sample sizes, resulting in non-significant findings for Th17 measurements. To address this limitation, future experiments will involve increased sample sizes to ensure more robust and conclusive results.

Our results confirm that *Ts*AES induces the expansion of Treg cells. In our study, immunization with *Ts*AES prior to DSS treatment led to a significant increase in Tregs within the cLP, as demonstrated by both flow cytometric analysis and immunofluorescence staining. Flow cytometry of mixed cells isolated from the cLP further validated the elevated number of Tregs. These findings indicate that *Ts*AES exhibits TGF-β-like activity and primarily promotes Treg differentiation from peripheral sources. Notably, while assessing the effects of *Ts*AES in vitro, we observed a marked induction of the anti-inflammatory cytokine IL-10. However, TGF-β levels were undetectable by ELISA, suggesting that alternative, more sensitive methods may be required for future evaluations.

*Ts*AES has been shown to alleviate IBD by suppressing Th1 cell-mediated secretion of pro-inflammatory factors while simultaneously promoting Th2 and Treg cells to produce anti-inflammatory cytokines. Preliminary laboratory studies suggest that *Ts*AES plays a pivotal role in modulating immune responses in autoimmune disorders such as IBD [19, 20]. Furthermore, existing literature indicates that tTregs can be safely applied in human therapies, and animal studies have demonstrated the therapeutic potential of pTregs. Our previous study identified *T. spiralis* paramyosin (*Ts*Pmy) as a key immunomodulatory protein capable of inducing Tregs [27]. Specifically, *Ts*Pmy promoted the expansion of tTregs rather than pTregs in the inflamed colon, enhancing the differentiation of effector Tregs (eTregs) with superior suppressive function and stability in colitis models [1]. In the present experiment, *Ts*AES was employed to stimulate splenic lymphocyte differentiation in vitro. The resulting Treg cells were isolated by flow cytometry and adoptively transferred via tail vein injection into mice to establish a DSS-induced acute colitis model. Significant weight loss occurred by the fifth day post-induction across all experimental groups, but no statistically significant differences in weight loss were observed among groups. This weight loss may be related to parasite antigens that increase insulin secretion, enhance lipid metabolism and elevate metabolic rates. Importantly, the DAI of the mice receiving *Ts*AES-induced Tregs was significantly lower than that of other groups. Flow cytometric analysis confirmed the presence of Tregs in both the *Ts*AES and PBS groups, suggesting that the observed Treg populations might result from either depletion of existing Tregs or their migration to other tissues. Further studies are necessary to clarify the underlying mechanisms involved.

Histological analysis using H&E staining revealed extensive infiltration of inflammatory cells in the colons of untreated mice with DSS-induced acute colitis. In contrast, mice that received reinfused Tregs showed a significant reduction in inflammatory cell infiltration and a restoration of normal tissue architecture. These results demonstrate that both *Ts*AES treatment and Treg reinfusion not only alleviate inflammation but also facilitate tissue repair. Notably, the beneficial clinical effect of Treg reinfusion derived from *Ts*AES stimulation was significantly greater than that observed in the PBS-induced Treg group, underscoring the potential of *Ts*AES to enhance Treg-mediated immunoregulatory mechanisms in colitis.

## Conclusions

Our study confirms that *Ts*AES exhibits TGF-β-like activity, promoting the induction of pTregs capable of alleviating IBD. *Ts*AES enhances the proliferation of pTregs within the colonic lamina propria, which correlates with increased levels of IL-10. These findings highlight the beneficial clinical effect of *Ts*AES in IBD treatment and provide further support for "helminth therapy" as an innovative approach to managing immune-mediated diseases. Ongoing clinical trials investigating helminth-derived proteins for a range of conditions, including IBD, allergic rhinitis, autism and food allergies, underscore the promising future of helminthic therapies in clinical applications.

## Data Availability

The data supporting the findings of the study must be available within the article and/or its supplementary materials, or deposited in a publicly available database.

## Abbreviations

Bregs: Regulatory B cells
CD: Crohn's disease
cLP: Colonic lamina propria
DAI: Disease activity index
DCs: Dendritic cells
DSS: Dextran sulfate sodium
ES: Excretory-secretory antigen
IBD: Inflammatory bowel disease
IL-2: Interleukin-2
LPMCs: Lamina propria mononuclear cells
pTregs: Peripheral regulatory T cells
TGF-β: Transforming growth factor-beta
Tregs: Regulatory T cells
*Ts*AES: *Trichinella spiralis* adult excretory-secretory antigen
tTregs: Thymus-derived regulatory T cells
UC: Ulcerative colitis
